# Prioritising Polysomnography in Children with Suspected Obstructive Sleep Apnoea: Key Roles of Symptom Onset and Sleep Questionnaire Scores

**DOI:** 10.3390/children11101228

**Published:** 2024-10-10

**Authors:** Luana Nosetti, Marco Zaffanello, Daniela Simoncini, Gaia Dellea, Maddalena Vitali, Hajar Amoudi, Massimo Agosti

**Affiliations:** 1 Pediatric Sleep Disorders Center, Division of Pediatrics, “F. Del Ponte” Hospital, University of Insubria, 21100 Varese, Italy; luana.nosetti@uninsubria.it (L.N.); daniela.simoncini@asst-settelaghi.it (D.S.); gdellea@studenti.uninsubria.it (G.D.); mvitali@studenti.uninsubria.it (M.V.); hamoudi@studenti.uninsubria.it (H.A.); 2 Department of Surgery, Dentistry, Pediatrics and Gynecology, University of Verona, 37100 Verona, Italy; 3 Woman and Child Department, Varese Hospital, Insubria University, 21100 Varese, Italy; massimo.agosti@uninsubria.it

**Keywords:** child, obstructive sleep apnoea, paediatric sleep questionnaire, polysomnography, sleep-disordered breathing, waiting time

## Abstract

Background/Objectives: Obstructive Sleep Apnoea Syndrome (OSA) in children disrupts normal breathing patterns and sleep architecture, potentially leading to severe consequences. Early identification and intervention are crucial to prevent these issues. This study explored the relationships between waiting times for polysomnography (PSG), clinical history, patient age at the time of PSG, and PSG outcomes in children. Methods: Two hundred and fourteen children were prospectively enrolled. Data were extracted from medical records regarding the patients’ age at the time of a referral for PSG and their age at the time of its execution. Information on the waiting times for PSG, a diagnosis (primary snoring, mild, moderate, and severe OSA), and a history of snoring and apnoea were also collected. Additional data included medications, surgical interventions, passive smoke exposure, and allergies. The records also included the Paediatric Sleep Questionnaire (PSQ). Results: The patient age at the time of a PSG prescription was lower for patients with a short history of sleep apnoeas (≤12 months; 4.6 (SD 2.6) years) compared to those with a long history (>12 months; 5.5 (SD 2.7) years; *p* = 0.027). The waiting time from prescription to PSG execution was shorter for patients with a short history of sleep apnoea (4.1 (SD 3.8) months) compared to those with a longer history (5.9 (SD 3.8) months; *p* = 0.001). A higher frequency of having an adenotonsillectomies before a PSG prescription was observed in the long-history group compared to the short-history group (13.3% vs. 6.9%). Conversely, a higher frequency of adenoidectomies before a PSG prescription was noted in the short-history group compared to the long-history group (9.7% vs. 1.3%). Conclusions: This study found that younger children with a shorter history of OSA are diagnosed and evaluated earlier than older children with a longer history of the condition, suggesting that prolonged symptoms may delay a diagnosis. ENT surgeries also vary among patients, with less invasive procedures (adenoidectomy) being more common in younger children with shorter histories of OSA. The increasing awareness of OSA highlights the need for improved access to diagnostic and treatment resources.

## 1. Introduction

Sleep-disordered breathing (SDB) encompasses a range of conditions. These include primary snoring, upper airway resistance syndrome, obstructive hypoventilation, and obstructive sleep apnoea (OSA). OSA is the most common manifestation of SDB [1]. OSA is a serious condition characterised by repeated blockages of the upper airway. These blockages lead to interrupted sleep patterns, fluctuations in blood oxygen levels, and episodes of hypoxia [2].

PSG is the preferred method for diagnosing OSA [3]. However, PSG is typically available only in specialised centres, limiting its widespread use in daily clinical practice [4]. The complexity of the test and the need for specialised expertise in diagnosing infants and young children present additional challenges. Social and economic barriers also contribute to delays in a diagnosis and treatment initiation [5]. Other methods, such as questionnaires, can assist in identifying potential cases of the disorder [6,7]. Specifically, the Paediatric Sleep Questionnaire (PSQ) is a well-known tool used to screen children aged two to eighteen for various sleep disorders, especially OSA [8].

SDB is fairly common among children, affecting about 2–4% of the population [9,10]. Snoring is reported in 3–15% of cases, while OSA occurs in 1–5%, most often in children aged two to six [1,11]. Despite these statistics, studies have not shown a clear link between gender and the severity of OSA [12].

Detecting paediatric OSA early is essential to avoiding complications later in life. However, a diagnosis is often delayed due to the time between noticing symptoms, seeking medical advice, and completing the diagnostic process [13,14,15]. Factors that can prolong this delay include low socioeconomic status, living in rural or underserved areas, occupational constraints, and personal traits such as awareness and self-efficacy. Paediatric OSA is frequently overlooked or underdiagnosed by both parents and healthcare providers [16].

Waiting times for PSG can vary significantly across the country, with no clear geographical patterns [17,18]. One key factor influencing these delays is access to healthcare services [18]. Patients from lower socioeconomic backgrounds often endure longer waits for PSG [18,19]. Reports from various countries highlight the substantial delays in diagnosing and treating SDB in children. In Belgium, wait times range from 2 to 36 months, while in Canada and the UK, they can be as long as 60 months [18,20].

This study aims to enhance the decision-making process for prescribing PSG. It also seeks to improve healthcare resource utilisation and reduce the diagnostic and treatment delays for children suspected of having OSA.

## 2. Materials and Methods

Inclusion and exclusion criteria:

### 2.1. Inclusion Criteria

The inclusion criteria included the following:

- Paediatric patients (up to 18 years of age) who were prescribed and subsequently underwent PSG for the evaluation of suspected SDB.
- A valid medical prescription for PSG to investigate symptoms of SDB, including snoring and apnoeas, must be documented.
- Availability of a detailed medical history concerning snoring, apnoeas, or other indicators of SDB prior to PSG prescription.
- Patients for whom the PSQ was administered during the PSG prescription process.
- Complete medical records that must include the following:
- Patient age at the time of PSG prescription and execution.
- The interval between PSG prescription and execution.
- PSG results, including parameters such as AHI, ODI, minimum SpO_2_ levels, and percentage of time spent snoring.
- A documented history of medications administered prior to PSG (oral, inhaled, or nasal spray).
- Record of any prior ENT surgeries (e.g., adenoidectomy, adenotonsillectomy) before PSG prescription (the protocol does not investigate the decision-making process behind surgical interventions).
- Information on passive exposure to tobacco smoke.
- Results of allergy testing (e.g., skin prick tests).

### 2.2. Exclusion Criteria

The exclusion criteria included the following:

- Patients with missing or incomplete data regarding PSG parameters, PSQ results, or medical history related to SDB.
- PSG reports lacking key results such as AHI, ODI, minimum SpO_2_ levels, or snoring percentage.
- Absence of PSQ administration at the time of PSG prescription.
- Incomplete or unclear medical history concerning snoring, apnoeas, or relevant medications prior to PSG prescription.
- Patients with no information on prior ENT surgeries relevant to sleep apnoea.
- Patients for whom the waiting time between PSG prescription and execution is not documented.
- Patients with missing or inconclusive allergy test results (e.g., prick test).

### 2.3. Key Investigation Points

In this study, we identified four primary areas of investigation:

- The presence of apnoeas and medical history of snoring prior to the PSG prescription.
- Patient age at the time of PSG prescription and the results of the PSQ, administered concurrently.
- The waiting time between PSG prescription and execution.
- Patient age at the time of PSG execution.

Specifically, we retrospectively collected the following data from medical records:

- Patient age (in years) at the time of PSG prescription.
- Waiting time (in months) between PSG prescription and execution.
- Age (in years) at the time of PSG execution.
- PSQ results recorded at the time of PSG prescription.
- PSG results captured at the time of execution, including the following:
- Apnoea–Hypopnea Index (AHI).
- Oxygen Desaturation Index (ODI).
- Minimum SpO_2_ (%).
- Percentage of time spent snoring.

The patient’s age at the time of PSG execution was categorised based on the severity of SDB into primary snoring, mild OSA, moderate OSA, and severe OSA.

Additional data collected included the following:

- A medical history of snoring and apnoeas prior to PSG prescription.
- Record of orally administered medications, such as corticosteroids, antihistamines, and antileukotrienes. Also included were medications administered via inhalers or nasal sprays, like salbutamol, prior to PSG prescription. This is essential for assessing comorbidities such as asthma and allergic rhinitis.
- Documented history of ENT surgeries performed before PSG prescription. The protocol did not investigate the decision-making process behind these surgical interventions.
- Exposure to passive smoke prior to PSG prescription.
- Documented allergies to inhalant allergens.

**Figure 1:**
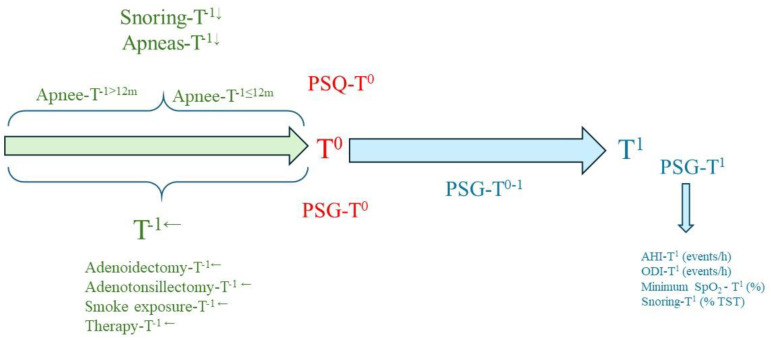
Study design. Legend: adenoidectomy-T^−1←^, history of adenoidectomy performed before PSG prescription; adenotonsillectomy-T^−1←^, history of adenotonsillectomy performed before PSG prescription; AHI-T^1^ (events/h), apnoea–hypopnea index results obtained at PSG-T^1^ (years); apnoeas-T^−1↓^, history of apnoeas before PSG prescription; apnee-T^−1≤12 m^, short history of sleep apnoeas (≤12 months); apnee-T^−1>12 m^, long history of sleep apnoeas (>12 months); minimum SpO_2_-T^1^ (%), minimum SpO_2_ (%) results obtained at PSG-T^1^ (years); ODI-T^1^ (events/h), oxygen desaturation index results obtained at PSG-T^1^ (years); PSG-T^0^ (years), patient age at the time of PSG prescription; PSG-T^0–1^ (months), waiting time from prescription to PSG execution; PSG-T^1^ (years), patient age at the time of PSG execution; PSQ-T^0^, results of PSQ administered at the same time as PSG-T^0^; smoke exposure-T^−1←^, smoke exposure; snoring-T^−1↓^, medical history of snoring before PSG prescription; snoring-T^1^ (% total sleep time, TST), snoring (%) results obtained at PSG-T^1^ (years); T^−1←^, clinical history of the symptom (snoring-T^−1^←, apnee-T^−1^←); T^1^, time at PSG execution; and therapy-T^−1←^, medical history of orally administered medications before PSG-T^0^.

Study design is shown in Figure 1.

### 2.4. Polysomnography

A comprehensive overnight PSG execution study was conducted using a Healthdyne Technologies device (E-series Compumedics) [21]. The recorded parameters included nasal pressure, airflow (thermistor), thoracic/abdominal movements, pulse oximetry, electrocardiogram, and transcutaneous carbon dioxide. Sleep staging adhered to the Kales and Rechtshaffen criteria, utilising electroencephalogram, electrooculogram, and submental electromyogram data. Additionally, PSG incorporated audio/video recordings and a body position sensor. Sleep stage scoring criteria were established based on the Kales and Rechtshaffen guidelines. OSA was identified when a thermistor airflow of at least 90% reduction persists for at least two breaths. On the other hand, obstructive hypopnea is marked by a decrease in airflow between 50% and 90%, accompanied by a minimum of 3% oxygen desaturation or an EEG arousal. Oxygen desaturation was considered at a ≥3% decrease from the baseline oxygen saturation. The AHI was computed as the total number of obstructive apnoeas and hypopneas divided by the total sleep time (TST) in hours. The PSG results were interpreted according to the AASM guidelines [22]. Based on the AHI results, the OSA was also categorised as mild (1 ≤ AHI ≤ 5 events/h), moderate (5 < AHI ≤ 10 events/h), or severe (AHI > 10 events/h) [12].

### 2.5. Paediatric Sleep Questionnaire

The results of the PSQ administered at the same time as PSG were used to screen for patient OSAand were completed by parents or caregivers. The PSG was validated for the Italian population [23]. It comprises 22 questions requiring a “yes”, “no”, or “do not know” response. The total score is determined by calculating the percentage of affirmative (“yes”) responses, with a significant result if the positive responses account for more than 33% of the overall count.

### 2.6. Statistical Analysis

The Kolmogorov–Smirnov test is typically employed to determine if a continuous variable adheres to a normal distribution. The Mann–Whitney test was utilised for a pair of values, yielding the means and standard deviations (SD) for a range of outcome measures. The chi-square test was used in the exploration of categorical variables. Nevertheless, the Fisher’s exact test was preferred when the values fell below 5. The contingency coefficient was also calculated, providing information on the strength of the association between variables. For variables reported as time intervals, the lower value was utilised. A bivariate correlation analysis was employed to calculate the Spearman correlation coefficient, expressed as r (*p*-value). The correlation between two variables, while adjusting for the confounding variables, was ascertained using the zero-order correlation in partial correlations. A multiple linear regression analysis was utilised to identify the predictive factors for a specified dependent variable. Predictors are various factors that may influence the dependent variable under study. The table will present unstandardised coefficients, associated T-scores, standard errors (S.E.), standardised coefficients (Beta), t-statistics, *p*-values, and 95% confidence intervals for coefficients. These data provide information on the strength and direction of the association between each predictor and the dependent variable and the statistical implication of these associations. A *p*-value < 0.05 was deemed statistically significant. Particularly in studies with smaller sample sizes, *p*-values between 0.05 and 0.1 should be interpreted cautiously, as they can introduce more significant uncertainty and elevate the risk of type I errors [24,25].

The data were entered into a Microsoft^®^ Excel^®^ (Version 2409) database on a Windows 11 system and then subjected to a statistical analysis using SPSS version 22.0 for Windows (SPSS Inc., Chicago, IL, USA).

This study complied with the Declaration of Helsinki, and the patients’ parents signed an informed consent form before this study was included. This clinical study was approved by the Insubria Ethical Committee (on 25 May 2019, study number 14/2019).

## 3. Results

Table 1 and Table 2 describes the statistical analyses performed on the continuous (Table 1) and categorical (Table 2) variables of the 214 enrolled paediatric patients in this study.

Table 3 compares the continuous variables between patients with a short history of sleep apnoeas (≤12 months) and those with a long history (>12 months) based on their age at the time of a PSG prescription.

Patients with a shorter history of sleep apnoea (≤12 months) had a mean apnoea duration of 7.35 months (SD 3.74) before a PSG prescription (Table 3). This duration was significantly shorter than the 23.2 months (SD 2.5) observed in those with a longer history (>12 months) (*p* = 0.001). Similarly, the mean duration of snoring was 15.2 months (SD 12.7) in the shorter history group, compared to 30.3 months (SD 10.8) in the longer history group (*p* = 0.001).

The patient age at the time of a PSG prescription was 4.6 years (SD 2.6) for patients with a short history of sleep apnoeas (≤12 months) and 5.5 years (SD 2.7) for those with a long history (>12 months). This difference was statistically significant (*p* = 0.027). The waiting time from prescription to PSG execution was 4.1 months (SD 3.8) for patients with a short history of sleep apnoeas (≤12 months) and 5.9 months (SD 3.8) for those with a long history (>12 months). This difference was also statistically significant (*p* = 0.001).

Additionally, the patient age at the time of the PSG execution was 4.9 years (SD 2.7) for patients with a short history of sleep apnoeas (≤12 months) and 6.0 years (SD 2.7) for patients with a long history (>12 months). This difference was statistically significant (*p* = 0.006).

None of the results of the patient age at the time of the PSG execution recording (AHI, ODI, SpO_2_ minimum, and snoring) differed between the short history of sleep apnoeas (≤12 months) group and the long-history (>12 months) group.

Table 4 presents the statistical analysis of the categorical variables. These include gender, PSG outcome categories, pharmacological therapy, otorhinolaryngologic surgery on the tonsils and adenoids, passive smoke exposure, and allergies. The analysis was conducted among patients with a short history of sleep apnoeas (≤12 months) and those with a long history of sleep apnoeas (>12 months).

The frequency distribution of otorhinolaryngologic surgeries was compared between the patients with a short history of OSA (≤12 months) and those with a long history of OSA (>12 months). This comparison included patients with a history of adenoidectomy and those with a history of adenotonsillectomy prior to a PSG prescription. Both comparisons reached statistical significance (*p*-value < 0.05). However, a higher frequency of adenotonsillectomy before a PSG prescription (13.3%) was observed in the long-history group (>12 months) compared to the short history of OSA group (6.9%). Additionally, a higher frequency of adenoidectomy before a PSG prescription (9.7%) was noted in the short history of OSA group (≤12 months) compared to the long history of OSA group (1.3%).

The percentage distribution of patient age at the time of the PSG execution derived SDB categories, medical history of medication before a PSG prescription categories (taken orally, nasally, or via inhalation), passive smoke exposure before a PSG prescription (*p* = 0.751), and allergies (*p* = 0.189) did not significantly differ between patients with a short history of sleep apnoeas (≤12 months) and those with a long history (>12 months). However, although the difference in allergy rates between the two groups did not reach statistical significance, it is noteworthy that the group with a longer history of apnoeas showed a higher prevalence of allergies (75% vs. 50%). This insignificant trend suggests a potential association between allergic conditions and the persistence of OSA symptoms.

Table 5 compares patients who underwent PSG within ≤3 months of waiting to those who had it performed after >3 months. The patient age (in years) at the time of a PSG prescription did not show a statistically significant difference between the two groups (*p* = 0.688). The mean age of the group with PSG that performed after >3 months was higher [5.8 (2.9) years] compared to the group with a waiting time of ≤3 months [5.1 (2.6) years]. However, this difference was only close to being statistically significant (*p* = 0.053).

In addition, the group that had PSG performed after >3 months of waiting had a longer mean duration of apnea history before a PSG prescription [17.7 (8.1) months] compared to the group with PSG performed within ≤3 months [11.6 (8.0) months; *p* < 0.001]. Similarly, the group with PSG performed after >3 months of waiting had a longer mean duration of snoring history before a PSG prescription [23.4 (13.8) months] compared to the group within ≤3 months [20.5 (14.2) months] of waiting, though this difference was not statistically significant (*p* < 0.084).

No statistically significant differences were found in the PSG outcome scores (AHI, ODI, and snoring) between the two groups at the time of execution (*p* = NS). Finally, the PSQ score, administered simultaneously with the PSG prescription, also did not show any statistically significant differences between the groups (*p* = 0.313).

Table 6 presents the statistical analysis of the categorical variables. These variables include gender, PSG result categories, pharmacological history of orally administered medications before patient age at the time of a PSG prescription, history of tonsil and adenoid surgeries performed before a PSG prescription, passive smoke exposure prior to a PSG prescription, and allergies. The analysis compares patients who underwent PSG within ≤3 months of waiting to those who had PSG performed after >3 months of waiting.

The categories of the PSG results (snoring, mild, moderate, and severe OSA) showed slight differences between the two groups, with the Pearson’s chi-square test yielding a *p*-value of 0.085. Specifically, the percentage of patients with snoring was higher in the group that had PSG performed after >3 months of waiting (16.2%) compared to those who had PSG performed within ≤3 months (7.7%). Conversely, the percentage of combined moderate and severe OSA was higher in the group who underwent PSG within ≤3 months (50%) compared to those who had PSG performed after >3 months (34.6%).

Finally, all other categorical variables—gender, medication, surgery, passive smoke exposure, and allergies—were comparable between the groups who underwent PSG within ≤3 months and those who had PSG performed after >3 months of waiting.

Table 7 presents the Spearman correlation coefficients and their corresponding two-tailed significance values for various correlated variables.

Patient age at the time of a PSG prescription was positively correlated with a history of snoring before the PSG prescription (r = 0.216, *p* = 0.003), a history of apnoea before the PSG prescription (r = 0.187, *p* = 0.023), and the results of the PSQ (score) administered at the time of the PSG prescription (r = 0.167, *p* = 0.015). In contrast, patient age at the time of a PSG prescription was negatively correlated with the ODI (r = −0.152, *p* = 0.026). After adjusting for their age at the time of the PSG execution, patient age at the time of a PSG prescription was negatively correlated with their history of apnoea before the PSG prescription (r = −0.290, *p* = 0.001).

Patient age at the time of the PSG execution was positively correlated with a history of snoring before a PSG prescription (r = 0.233, *p* = 0.001), a history of apnoea before the PSG prescription (r = 0.237, *p* = 0.004), and the PSQ score administered at the time of the PSG prescription (r = 0.143, *p* = 0.037). It was negatively correlated with the ODI (r = −0.162, *p* = 0.018). Additionally, when adjusted for patient age at time of a PSG prescription, their age at the time of the PSG execution was positively correlated with a medical history of snoring before the PSG prescription (r = 0.189, *p* = 0.027) and a history of apnoea before the PSG prescription (r = 0.308, *p* < 0.001).

The waiting time (in months) from prescription to PSG execution was positively correlated with a history of apnoea before the PSG prescription (r = 0.327, *p* < 0.001). After adjusting for patient age at the time of a PSG prescription, waiting time (in months) was positively correlated with a history of snoring before a PSG prescription (r = 0.195, *p* = 0.023) and a history of apnoea before the PSG prescription (r = 0.259, *p* = 0.002). It was also negatively correlated with the PSQ (score) results administered at the time of the PSG prescription (r = −0.172, *p* = 0.045). After adjusting for age at the time of the PSG execution, the waiting times (in months) remained positively correlated with a history of apnoea before the PSG prescription (r = 0.241, *p* = 0.004) and negatively correlated with the PSQ (score) results administered at the same time as the PSG prescription (r = −0.179, *p* = 0.036).

No statistically significant correlation was found between patient age at the time of a PSG prescription and AHI (r = −0.116, *p* = 0.091). Similarly, no significant correlation was observed between patient age at the time of a PSG prescription and the percentage of total sleep time with snoring (%TST) (r = 0.086, *p* = 0.210). Likewise, no significant correlation was found between patient age at the time of the PSG execution and AHI (r = −0.127, *p* = 0.064) or snoring %TST (r = −0.075, *p* = 0.273).

The waiting times between a PSG prescription and its execution did not show a relevant correlation with AHI (r = −0.097, *p* = 0.157) or snoring %TST (r = −0.045, *p* = 0.512). Even after adjusting for patient age at the time of a PSG prescription, the results did not indicate any significant correlation with AHI (r = −0.005, *p* = 0.957), ODI (r = −0.004, *p* = 0.959), or snoring %TST (r = −0.009, *p* = 0.916). Finally, after adjusting for patient age at the time of the PSG execution, no significant correlations were found between snoring %TST (r = −0.017, *p* = 0.841), AHI (r = −0.032, *p* = 0.714), or ODI (r = −0.020, *p* = 0.813) and the waiting times.

The variables investigated in Table 8 could most readily influence the waiting times for PSG execution. The linear regression analysis revealed three significant predictors of waiting times from PSG prescription to PSG execution: a history of apnoea before the PSG prescription, the PSQ score at the time of the PSG execution, and a history of an adenoidectomy. The positive and significant coefficient of the predictor “history of apnoea before PSG prescription” indicates that an increase in a medical history of apnoea (in months) is associated with a 0.133-unit increase in the dependent variable, waiting time (in months), from prescription to PSG execution. The results of the PSQ administered at the same time as the PSG prescription have an unstandardised coefficient of −3.327. This suggests that a one-unit increase in the PSQ score is associated with a decrease of 3.3 units in waiting time (in months) from PSG prescription to PSG execution. Furthermore, the presence of a history of adenoidectomy is related to an increase of 3.349 months in the waiting time to perform the PSG.

## 4. Discussion

This study investigated the relationship between a history of OSA, patient age at the time of a PSG prescription, and PSG results in children. Children with shorter OSA histories (≤12 months) underwent PSG prescriptions and evaluations at younger ages (4.6 (2.6) years and 4.9 (2.7) years, respectively) compared to those with longer histories (5.5 (2.7) years and 6.0 (2.7) years, respectively). This trend may reflect the delayed diagnoses in patients with prolonged symptoms. Prolonged waiting times for PSG were correlated with longer histories of apnoea (5.9 (3.8) months vs. 4.1 (3.8) months; *p* = 0.001), potentially hindering a timely diagnosis and treatment. However, no significant differences were observed in the PSQ scores between the groups. The children with shorter waiting times exhibited comparable OSA severity (AHI 7.7 (8.5) events/h) to those with longer waits (AHI 6.6 (7.6) events/h; *p* = 0.226), highlighting the importance of timely evaluations. Younger children with a shorter history of apnoea, and similar PSQ scores to those with a prolonged history of apnoea, were prioritised for PSG, indicating effective referral strategies. The children with shorter histories of apnoea often underwent less invasive surgeries, such as an adenoidectomy, while those with longer histories required more extensive procedures, such as an adenotonsillectomy. Factors such as passive smoke exposure and medication use did not significantly influence surgical choices. The increasing awareness of OSA has led to a rising demand for sleep laboratory resources, highlighting the need for more accessible and cost-effective ambulatory methods to diagnose and manage OSA [26,27]. Such measures are essential to meet the growing demand for sleep services and effectively manage the disease’s increasing burden [26].

Children with a shorter history of apnoea tend to be younger when PSG is prescribed and performed. They also experience shorter waiting times compared to children with a more extended history of apnoea. Clinical suspicion of OSA may arise based on symptoms, but PSG is essential for a definitive diagnosis [28]. Delays in a diagnosis are often influenced by the availability of sleep centres and diagnostic tools [6]. Sleep studies conducted in outpatient settings can promote the early detection and management of OSA, potentially leading to significant cost savings. This approach could serve as a viable alternative to the traditional in-laboratory sleep studies, which may not be as accessible or affordable for all patients [29].

Unexpectedly, children with more recent symptoms of apnoea (≤12 months) are identified and referred for PSGs more quickly (*p* = 0.027), resulting in shorter waiting times (4.1 (3.8) months vs. 5.9 (3.8) months; *p* = 0.001). This may be due to the increased awareness among healthcare providers and parents regarding the importance of the early detection of OSA in children.

Additionally, children with a more prolonged history of OSA showed similar levels of passive smoke exposure compared to those with shorter histories (*p* = 0.751). This suggests that smoke exposure, regardless of duration, may have a comparable impact on OSA severity. Studies have also linked passive smoking to the development of OSA in children [30,31,32].

Research has shown that the PSQ can be a valuable tool for assessing the severity of OSA in children and directing them to an appropriate treatment [33]. Early screening for OSA is crucial, and questionnaires like the PSQ effectively identify at-risk children. Those who screen positive should be referred to specialised centres for further evaluation using PSG [34]. Given the limited availability of PSG, initial screening tools such as the PSQ play a vital role in ensuring timely and appropriate care [6]. However, some studies suggest a lack of correlation between PSQ scores and PSG results, indicating that the PSQ may not always be a reliable screening tool [33]. Therefore, while a medical history alone may not be sufficient, a combination of a clinical assessment, a history review, questionnaires, and objective measurements are essential for a comprehensive evaluation [35].

Children who experienced shorter waiting times for PSG (≤3 months) tend to have a higher prevalence of moderate to severe OSA compared to those with prolonged waiting times (>3 months), although this difference did not reach statistical significance (*p* = 0.085). This may be due to more noticeable symptoms or a higher clinical suspicion of OSA in these cases [33].

Additionally, children with longer waiting times for PSG (>3 months) tended to have a longer history of snoring before a PSG prescription (*p* = 0.084) and were generally older at the time of the PSG (*p* = 0.053), although neither reached statistical significance. Furthermore, patient age at the time of a PSG prescription was positively correlated with the child’s history of snoring (r = 0.216; *p* = 0.003), history of apnoea (r = 0.187; *p* = 0.023), and their PSQ score (r = 0.167; *p* = 0.015), while being negatively correlated with the ODI (r = −0.152; *p* = 0.026). No differences were found regarding the PSQ score (*p* = 0.466), and final PSG results expressed as AHI, ODI, and snoring, between those with a brief and those with a longer history of apnoeas.

Research also confirmed that waiting times for PSG can extend from several months to over a year, potentially contributing to the underdiagnosis of OSA [36]. However, both shorter and longer waiting times for PSG showed similar average PSQ scores (*p* = 0.313), despite the PSQ being highly regarded for screening OSA in suspected cases [33].

An adenotonsillectomy was more frequent in children with a longer history of apnoea, whereas an adenoidectomy was more common in those with a shorter history. We hypothesise that children with a longer history of apnoea undergo an adenotonsillectomy more frequently due to the chronicity and severity of their condition. Additionally, children with a longer history of apnoea but milder PSG outcomes may still undergo an adenotonsillectomy, reflecting a tendency towards more aggressive treatment in chronic cases. Studies confirm that an adenotonsillectomy is the first-line treatment for paediatric OSA, significantly improving polygraphic [37] or PSG [38] outcomes. This procedure substantially impacts the upper airway and respiratory system [39].

Children with a shorter history of apnoea are more likely to undergo an adenoidectomy as an initial treatment. Although this may be a less invasive option, the potential for residual symptoms may be higher with an adenoidectomy compared to an adenotonsillectomy [40]. Unfortunately, the medical history provided by parents or caregivers does not allow for a distinction between a tonsillotomy and tonsillectomy in our sample. Both procedures are effective in treating OSA caused by enlarged tonsils. However, a tonsillotomy carries a slightly lower risk of post-surgical complications (2.6%) compared to a tonsillectomy (4.9%) [41].

A triage algorithm based on tonsil size was developed by Heath et al. for the management of paediatric OSA. Drug-Induced Sleep Endoscopy (DISE) has become an essential tool, providing critical insights that guide both surgical and non-surgical treatments [42]. Brodsky’s study recommended referring children with OSA and grade 2, 3, or 4 tonsils to an otolaryngologist. In contrast, children with smaller tonsils were referred to a respiratory paediatrician to streamline the referral process and reduce waiting times [43]. In Italy, a multidisciplinary expert group introduced an age-specific, stepped approach for diagnosing and managing paediatric SDB. This approach aims to ensure a timely and effective diagnosis and treatment for children with SDB, regardless of age [44]. However, when symptoms persist after an adenoidectomy or adenotonsillectomy, a follow-up PSG is often required. This follow-up is necessary to evaluate the effectiveness of the treatment.

The increased awareness of the long-term impacts of paediatric SDB has led to more consultations with paediatric respiratory sleep specialists, which, in turn, has extended waiting lists. Healthcare providers should recognise that children exhibit diverse OSA symptoms depending on their developmental stages [16,43]. By incorporating electronic questionnaires for uncomplicated cases, clinics can streamline processes and reduce waiting times for children referred for an SDB evaluation [45].

The strengths of this study include the large sample size of children with OSA and the examination of various factors affecting waiting times and PSG outcomes using appropriate statistical methods. However, the retrospective design limits the ability to establish clear cause-and-effect relationships. Furthermore, although this study considers several factors influencing waiting times and PSG results, the findings may not be generalisable to other populations. Another limitation is that this study did not assess the impact of waiting times for PSG on the long-term outcomes for children with OSA.

## 5. Conclusions

This study found that younger children with a shorter history of OSA are diagnosed and evaluated earlier than older children with longer histories of the condition. This suggests that prolonged symptoms may delay a diagnosis. Despite the comparable severity of OSA across different historical lengths, longer waiting times for PSG are linked to extended histories of apnoea. This underscores the importance of timely evaluations. The prioritisation of younger children with shorter histories of PSG indicates effective referral strategies. ENT surgeries also differ among patients, with less invasive procedures, such as an adenoidectomy, being more common among younger children with shorter histories of OSA. Overall, the rising awareness of OSA highlights the need for improved access to diagnostic and treatment resources.

Further research is needed to determine the most effective strategies for reducing PSG waiting times for PSG in children.

## Figures and Tables

**Table 1:** Summarises the continuous variables of the enrolled paediatric patients (n 214) in this study.

Continuous Variable	Mean (SD)	25° pc	50° pc	75° pc	Kolmogorov–Smirnov Test *
PSG prescription, age (years)	5.2 (2.8)	3.3	4.6	6.4	<0.001
PSQ administered at the same time as PSG prescription (score)	0.34 (0.19)	0.22	0.36	0.45	0.012
Waiting time from PSG prescription to execution (months)	5.2 (5.0)	2.1	4.2	8.2	<0.001
PSG execution, age (years)	5.6 (2.8)	3.8	5.0	6.8	<0.001
Weight (kg)	22.5 (13.7)	15	18	25	<0.001
Height (cm)	113 (19)	99	110	125	0.005
Results of the PSG					
AHI (events/h)	7.0 (7.9)	2.3	4.1	8.0	<0.001
ODI (events/h)	6.0 (7.4)	2	3.5	6.3	<0.001
SpO_2_ (%)	96.8 (1.0)	96.1	96.9	97.5	0.001
Minimum SpO_2_ (%)	89.1 (4.4)	88	90	92	<0.001
Snoring (%)	4.5 (4.6)	9	3.5	6.6	<0.001

Legend: *, All variables in this table are not normally distributed. AHI, apnoea–hypopnea index; ODI, oxygen desaturation index; PSG, polysomnography; PSQ, paediatric sleep questionnaire; and SD, standard deviation.

**Table 2:** Summarises the categorical variables of the enrolled paediatric patients in this study.

Categorical Variable	Subcategories	N (%)
Sex	Male	133 (62.1)
History of snoring before PSG prescription (minimum, months; missing = do not know)	I do not know	21 (9.8)
	0–6	15 (7)
	6–12	38 (17.8)
	12–18	4 (1.9)
	18–24	109 (50.9)
	>24	27 (12.6)
History of apnoeas before PSG prescription (minimum, months; missing = do not know)	I do not know	71 (33.1)
	0–6	18 (8.4)
	6–12	30 (14)
	12–18	29 (13.6)
	18–24	5 (2.3)
	>24	66 (30.8)
Drugs taken orally before PSG prescription	Negative	186 (86.9)
	Antihistamines	10 (4.7)
	Corticosteroids	5 (2.3)
	Antileukotrienes	4 (1.9)
	Other drugs	4 (1.9)
History of drugs spray and inhalants taken before PSG prescription	Negative	162 (75.7)
	Steroids	51 (23.8)
	Salbutamol	0
	Steroids, salbutamol	1 (0.5)
ENT surgery before PSG prescription	Adenotonsillectomy	15 (7.5)
	Adenoidectomy	9 (4.2)
	Tonsillectomy	1 (0.5)
Second-hand smoke exposure before PSG prescription	Positive	42 (19.6)
Allergy test positivity (prick test)	Yes	38 (17.8)
	Not performed	154 (72.0)
Results of PSG at execution (Categories)	Primary snoring	28 (13.1)
	Mild	100 (46.7)
	Moderate	43 (20.1)
	Severe	43 (20.1)

Legend: PSG, polysomnography.

**Table 3:** Comparison of continuous variables between patients whose history of sleep apnoeas are ≤12 months (short history) and >12 months (long history) after the medical prescription of polysomnography.

	Short History of Sleep Apnoeas (≤12 Months) (n. 72), Mean (SD)	Long History of Sleep Apnoeas (>12 Months) (n. 75), Mean (SD)	Mann–Whitney U Test, *p*
History of apnoea before PSG prescription (months)	7.35 (3.74)	23.2 (2.5)	0.001 *
History of snoring before PSG prescription (months)	15.2 (12.7)	30.3 (10.8)	0.001 *
Age at time of PSG prescription (years)	4.6 (2.6)	5.5 (2.7)	0.027 *
Results of PSQ administered at the same time as PSG prescription (score)	0.37 (0.2)	0.34 (0.21)	0.466
Waiting time from PSG prescription to execution (months)	4.1 (3.8)	5.9 (3.8)	0.001 *
Age at time of PSG execution (years)	4.9 (2.7)	6.0 (2.7)	0.006 *
AHI (events/h)	7.9 (8.2)	6.8 (7.5)	0.224
ODI (events/h)	6.7 (7.6)	6.0 (7.0)	0.316
SpO_2_ minimum (%)	87.9 (5.3)	89.3 (4.3)	0.127
Snoring (% TST)	4.3 (4.2)	4.3 (4.4)	0.946

Legend: *, statistically significant; AHI, apnoea–hypopnea index; ODI, oxygen desaturation index; PSG, polysomnography; PSQ, paediatric sleep questionnaire; SD, standard deviation; and TST, total sleep time.

**Table 4:** Comparison of categorical variables between patients (n = 147; n = 67 non-responders) who experienced the onset of sleep apnoea less than 12 months and more than 12 months prior to a prescription for polysomnography.

Categorical Variables	Subcategories	Short History of Sleep Apnoeas (≤12 Months), n. 72; %	Long History of Sleep Apnoeas (>12 Months), n. 75; %	Pearson Chi-Square (*p*-Value)	Fisher’s Exact Test (*p*-Value)	Contingency Coefficient
Sex	Males	63.9	60.0	0.647	-	0.040
History of adenoidectomy before PSG prescription	Yes	9.7	1.3		0.027 *	0.182
Tonsillectomy before PSG prescription	Yes	0	0	-	-	-
Adenotonsillectomy before PSG prescription	Yes	6.9	13.3	-	0.157	0.105
				0.012 *	-	0.238
Second-hand smoke before PSG prescription	Yes	16.7	18.7	0.751	-	0.026
Allergy	Yes	50.0	75.0	-	0.189	0.227
Drugs taken orally before PSG prescription	Antihistamines	2.8	2.7			
	Steroids	2.8	4.0			
	Antileukotrienes	1.4	4.0			
	Other	2.8	0.0	0.530	-	0.145
Drugs spray or inhalants taken before PSG prescription	Steroids	29.2	25.3			
	Salbutamol	0	0			
	Steroids + salbutamol	0	1.3	0.550		0.090
PSG result (Categories, %) ^§^	Snoring	9.7	10.7			
	Mild OSA	41.7	46.7			
	Moderate OSA	22.2	21.3			
	Severe OSA	26.4	21.3	0.885	-	0.066

Legend: *, statistically significant (*p* < 0.05); ^§^, mild OSA = 1 ≤ AHI ≤ 5 events/h, moderate OSA = 5 < AHI ≤ 10 events/h, or severe OSA = AHI > 10 events/h; OSA, obstructive sleep apnoea; and PSG, polysomnography.

**Table 5:** Comparison between patients who underwent polysomnography within 3 months of receiving a medical prescription and those who underwent PSG after 3 months of receiving a medical prescription.

	PSG within ≤3 Months of Waiting (n. 78); Mean (SD)	PSG Performed after >3 Months of Waiting (n. 136); Mean (SD)	Mann–Whitney Test (*p*-Value)
History of snoring before PSG prescription (minimum, months; missing = do not know)	20.5 (14.2) [Total Responses n. 74]	23.4 (13.8) [Total Responses n. 120]	0.084
History of apnoea before PSG prescription (minimum, months; missing = do not know)	11.6 (8.0) [Total Responses n. 55]	17.7 (8.1) [Total Responses n. 92]	<0.001 *
Age at time of PSG prescription (years)	5.0 (2.6)	5.3 (2.9)	0.688
Waiting time from PSG prescription to PSG execution	1.4 (1.0)	7.4 (5.1)	<0.001 *
Results of PSQ administered at the same time as PSG prescription (score)	0.35 (0.18)	0.34 (0.20)	0.313
Age at time of PSG execution (years)	5.1 (2.6)	5.8 (2.9)	0.053
AHI (Events/h)	7.7 (8.5)	6.6 (7.6)	0.226
ODI (Events/h)	6.2 (7.4)	5.8 (7.4)	0.361
Snoring (% TST)	4.6 (4.7)	4.5 (4.5)	0.751

Legend: *, statistically significant (*p* < 0.05); AHI, apnoea–hypopnea index; ODI, oxygen desaturation index; PSG, polysomnography; PSQ, paediatric sleep questionnaire; SD, standard deviation.

**Table 6:** Comparison of categorical variables (Pearson’s chi-square or Fisher’s exact test) between patients who had a waiting time for PSG of ≤3 months after a medical prescription for PSG and those who underwent PSG >3 months after a medical prescription for PSG.

Categorical Variables	Subcategories	PSG within ≤3 Months of Waiting, %	PSG Performed after >3 Months of Waiting; %	Pearson Chi-Square (*p*-Value)	Fisher’s Exact Test (*p*-Value)	Contingency Coefficient
N.	-	78	136	-	-	-
Sex	Males	64.1	61	0.656	0.383	0.030
PSG results (Categories)	Snoring	7.7	16.2			
	Mild OSA	42.3	49.3			
	Moderate OSA	26.9	16.2			
	Severe OSA	23.1	18.4	0.085	-	0.173
Medications were taken orally before PSG prescription	Antihistamines	7.7	2.9			
	Steroids	3.8	1.5			
	Antileukotrienes	0	2.9			
	Other	2.6	1.5	0.174	-	0.170
History of pharmacy Nasal Spray before PSG prescription	Steroids	26.6	22.8			
	Salbutamol	0	0			
	Steroids + salbutamol	0	0.7	0.680	-	0.060
History of adenotonsillectomy before PSG prescription	Yes	10.3	5.9	0.242	0.183	0.080
History of adenoidectomy before PSG prescription	Yes	3.8	4.4	0.843	0.573	0.014
Medical history of tonsillectomy performed before PSG prescription	No	100	99.3	0.448	0.636	0.052
				0.588		0.094
History of exposure to Second-hand smoke before PSG prescription	Yes	17.9	20.6	0.640	0.390	0.032
Allergy	Yes	68.4	61	0.578	0.398	0.072

Legend: OSA, obstructive sleep apnoea; PSG, polysomnography.

**Table 7:** The table presents the results of the Spearman’s correlation analysis between patient age at the time of PSG prescription, patient age at the time of PSG execution, waiting time from prescription to PSG execution, and their history of SDB, along with the results of the PSN and PSQ.

	N	Age at Time of PSG Prescription, Years (SD)	Age at Time of PSG Execution, Years (SD)	Waiting Time from Prescription to PSG Execution, Months (SD)
Age, years (SD)		5.2 (2.8)	5.6 (2.8)	5.2 (5.0)
Spearman’s correlation		r (two tail *p*-value)	r (two tail *p*-value)	r (two tail *p*-value)
History of snoring before PSG prescription (minimum, months; missing = do not know)	194	0.216 ^§^ (0.003 *)	0.233 ^§^ (0.001 *)	0.119 (0.098)
History of apnoea before PSG prescription (minimum, months; missing = do not know)	147	0.187 ^§^ (0.023 *)	0.237 ^§^ (0.004 *)	0.327 (<0.001 *)
Results of PSQ administered at the same time as PSG prescription (score)	214	0.167 ^§^ (0.015 *)	0.143 ^§^ (0.037 *)	−0.091 (0.186)
AHI (events/hour)	214	−0.116 (0.091)	−0.127 (0.064)	−0.097 (0.157)
ODI (events/hour)	214	−0.152 ^§^ (0.026 *)	−0.162 ^§^ (0.018 *)	−0.089 (0.195)
History of snoring before PSG prescription (% TST)	194	0.086 (0.210)	−0.075 (0.273)	−0.045 (0.512)
Spearman’s correlation; adjusted for age at the time of PSG prescription	214	r (two-tail *p*-value)	r (two-tail *p*-value)	r (two-tail *p*-value)
History of snoring before PSG prescription (minimum, months; missing = do not know)	214	-	0.189 ^§^ (0.027 *)	0.195 ^§^ (0.023 *)
History of apnoea before PSG prescription (minimum, months; missing = do not know)	214	-	0.308 ^§§^ (<0.001 *)	0.259 ^§^ (0.002 *)
Results of PSQ administered at the same time as PSG prescription (score)	214	-	−0.103 (0.230)	−0.172 ^§^ (0.045 *)
AHI (events/hour)	214	-	−0.005 (0.957)	−0.052 (0.549)
ODI (events/hour)	214	-	−0.004 (0.959)	−0.041 (0.634)
Russamento (% TST)	214	-	−0.009 (0.916)	−0.004 (0.962)
Spearman’s correlation, adjusted for age at the time of PSG execution	214	r (two tail *p*-value)	r (two tail *p*-value)	r (two tail *p*-value)
History of snoring before PSG prescription (minimum, months; missing = do not know)	214	−0.158 (0.066)	-	0.166 (0.052)
History of apnoea before PSG prescription (minimum, months; missing = do not know)	214	−0.290 ^§^ (0.001 *)	-	0.241 ^§^ (0.004 *)
Results of PSQ administered at the same time as PSG prescription (score)	214	0.111 (0.196)	-	−0.179 ^§^ (0.036 *)
AHI (events/hour)	214	−0.019 (0.830)	-	−0.032 (0.714)
ODI (events/hour)	214	−0.019 (0.822)	-	−0.020 (0.813)
Snoring (% TST)	214	0.024 (0.781)	-	−0.017 (0.841)

Legend: *, statistically significant (*p* < 0.05); ^§^, slight correlation; ^§§^, moderate correlation; AHI, apnoea–hypopnea index; ODI, oxygen desaturation index; PSG, polysomnography; PSQ, paediatric sleep questionnaire; SD, standard deviation; TST, total sleep time. Orange indicates a statistically significant (*p* < 0.05) positive correlation, while the colour green indicates a statistically significant (*p* < 0.05) negative correlation.

**Table 8:** A linear regression analysis to identify predictive factors for the waiting times for PSG.

Dependent Variable: Waiting Time to Perform PSG, Months (SD). Predictors: Age at PSQ Administration at the Same Time as PSG Prescription, Years (SD); History of OSA before PSG Prescription; History of Snoring before PSG Prescription (%); Results of PSQ Administered at the Same Time as PSG Prescription; Adenoidectomy; Adenotonsillectomy	Non-Standardised Coefficients, T	S.E.	Standardised Coefficients, Beta	t	*p*-Value	C.I. per B 95% (Lower–Upper)
Variables Entered in the Model						
History of apnoea before PSG prescription (Months)	0.133	0.036	0.264	3.647	<0.001 *	0.061–0.205
Results of PSQ administered at the same time as PSG prescription	−3.327	1.523	−0.167	−2.185	0.031 *	−6.339–(−0.316)
Adenoidectomy	3.349	1.338	0.204	2.503	0.014	0.702–5.996
Constant	3.8018	0.848	-	4.505	<0.001 *	2.142–5.495

Legend: *, statistically significant (*p* < 0.05); AHI, apnoea–hypopnea index; C.I., confidence interval; ODI, oxygen desaturation index; PSG, polysomnography; PSQ, paediatric sleep questionnaire; SD, standard deviation.

## Data Availability

Data are contained within the article.
